# Osteosarcoma of the distal radius treated by en bloc resection and reconstruction with a fibular shaft preserving the radiocarpal joint: A case report

**DOI:** 10.3892/ol.2014.1891

**Published:** 2014-02-18

**Authors:** XIUCHUN YU, SONGFENG XU, MING XU, YE YUAN

**Affiliations:** Department of Orthopedics, The General Hospital of Jinan Military Commanding Region, Jinan, Shandong 250031, P.R. China

**Keywords:** osteosarcoma, distal radius, reconstruction, fibular shaft

## Abstract

Osteosarcoma is the most common primary malignant bone tumor. The distal radius is a relatively common skeletal site for primary bone tumors, but not for osteosarcoma. It is difficult to treat Osteosarcoma of the distal radius; however, skeletal reconstruction and functional restoration following en bloc resection may be a promising technique. This report presents a 17-year-old male with osteosarcoma of the distal radius that was treated by en bloc resection and reconstruction using a fibular shaft to preserve the radiocarpal joint. After six months, radiographs revealed that the grafted fibular bone had healed well with the host bone. Physical examination demonstrated that active dorsiflexion of the affected wrist was to 90° and palmer flexion was to 45°. Fourteen months after surgery there was no evidence of wrist deformity, instability, metastasis or local recurrence. Therefore, this technique preserved the important structures and the joint surface for wrist stability and effective function.

## Introduction

Osteosarcoma is the most common primary malignant bone tumor of obscure origin, which generally presents in the second decade of life (1). The distal radius is a relatively common skeletal site for primary bone tumors, however, not for osteosarcoma; it was reported that <1% of osteosarcomas arise in the distal radius (2). En bloc resection of the distal radius is the mainstream treatment for malignant lesions and aggressive benign lesions (3). This poses a dual limitation of skeletal reconstruction and functional restoration due to the high functional demands of the hand, the long life expectancy of the patients, particularly for osteosarcoma treated with neoadjuvant chemotherapy and limb salvage, and the limited amount of surrounding soft tissue as well as the proximity of the adjacent nerves and tendons. The various procedures described include arthrodesis using bulk autograft (4), ulnar translocation (5), reconstruction with non-vascularized or vascularized fibular grafts (6,7), osteoarticular allograft (8) and prosthetic replacement (9). En bloc resection of the distal radius destroys the structure of distal radioulnar articulation and radiocarpal joint and results in the postoperative instability of radiocarpal articulation (10,11). This report presents a 17-year-old male with an osteosarcoma of the distal radius, which was treated by en bloc resection and reconstructed with a fibular shaft, thus preserving the radiocarpal joint. This technique enables a biological reconstruction with a precise anatomical fit, and avoids long-term endoprosthetic complications and the need for maintenance of bone-banking facilities for allografts. To the best of our knowledge, this report is the first to describe a case of osteosarcoma of the distal radius treated with this technique.

## Case report

**Figure 1:**
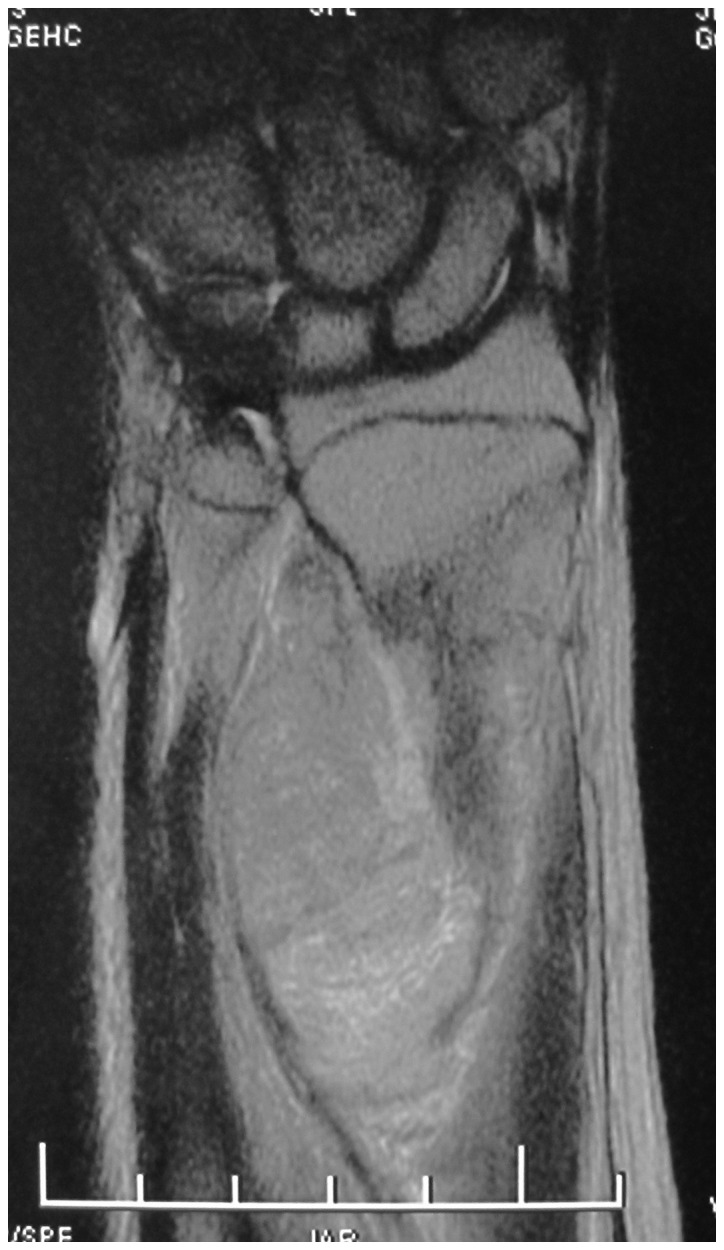
MRI revealed no epiphyseal invasion.

**Figure 2:**
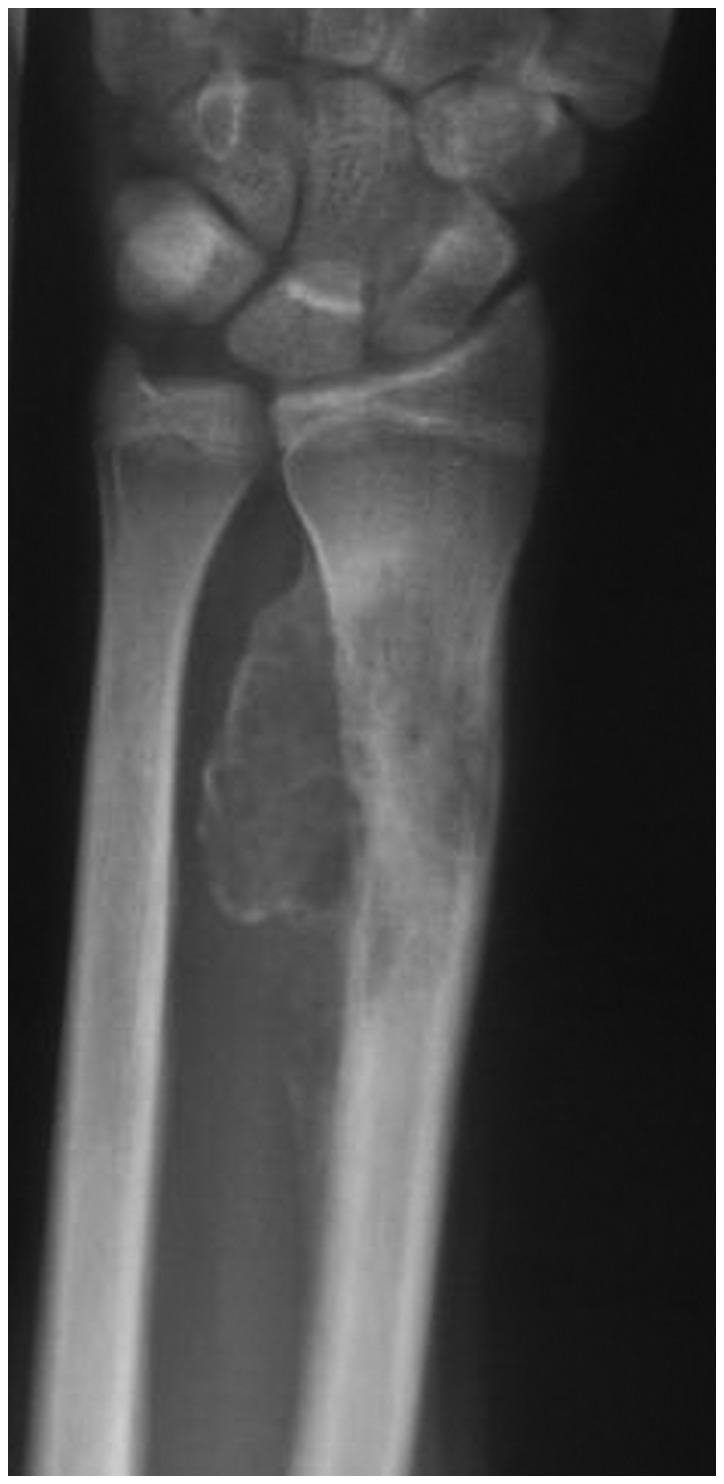
Plain radiography revealed sclerotic changes and a good lesion margin.

A 17-year-old male complained of mild right wrist pain for approximately two months. Physical examination at the Department of Orthopedics, The General Hospital of Jinan Military Commanding Region (Jinan, China) revealed diffuse swelling, mild tenderness and local heat at the distal end of the right radius. A hard mass with an irregular surface (size, 3×2 cm) was palpated in the dorsal aspect of the distal radius. Wrist motion was slightly restricted (extension, 55° and flexion, 45°). Radiographs showed diffuse osteosclerosis and focal osteolysis with periosteal reaction in the distal radius metaphysis. Magnetic resonance imaging (MRI) revealed a hypointense lesion on T1-weighted images and a hyperintense lesion on T2-weighted images with soft tissue extension. No epiphyseal involvement was identified on the MRI (Fig. 1). Technetium-99m scintigraphy showed an increased isotope uptake within the lesion. A chest computed tomography (CT) scan revealed no abnormalities. Laboratory data, including blood cell counts, C-reactive protein levels, erythrocyte sedimentation rate and serum alkaline phosphatase levels were within reference range. Urinalysis revealed no abnormalities. A needle aspiration biopsy was performed and histological examination of the specimen confirmed the diagnosis of osteosarcoma. Following two cycles, with an interval of three weeks, of the chemotherapy protocol with cisplatin (120 mg/m^2^ skin), adriamycin (90 mg/m^2^ of skin) and ifosfamide (10 g/m^2^ of skin), pain diminished, the local mass decreased and became rigid, and the range of motion of the affected wrist returned to normal. The sclerotic changes and a good margin of the lesion were observed on plain radiographs (Fig. 2), and MRI revealed marked shrinkage of the tumor as well as diminished marrow edema. According to the classification of musculoskeletal neoplasms by Enneking *et al* (11), the tumor was at surgical stage IIB.

**Figure 3:**
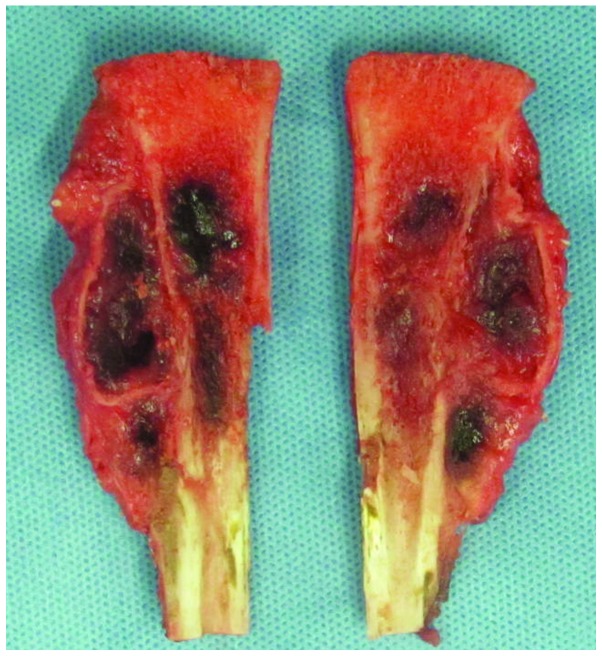
En bloc resection of the distal radial osteosarcoma.

**Figure 4:**
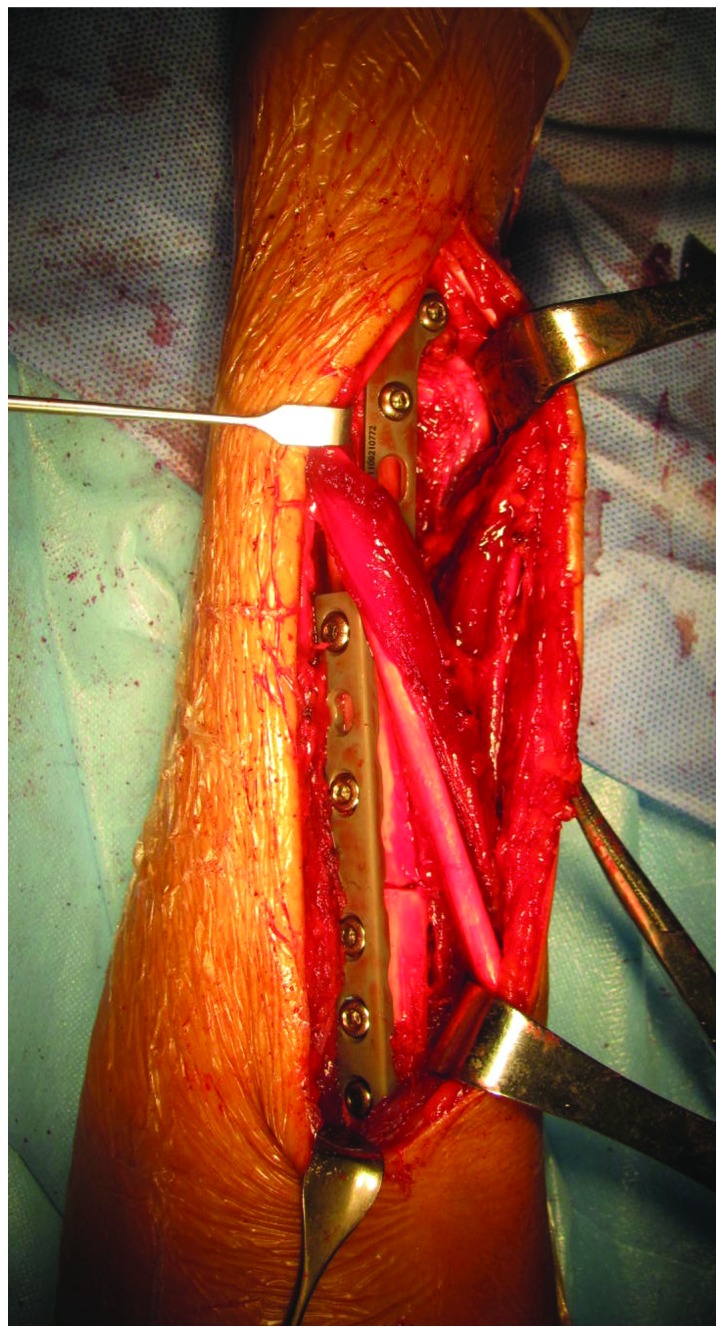
Ulna and distal radioulnar articulation, and radiocarpal joint were preserved. The free fibular shaft was fixed to the host bone with two plates.

**Figure 5:**
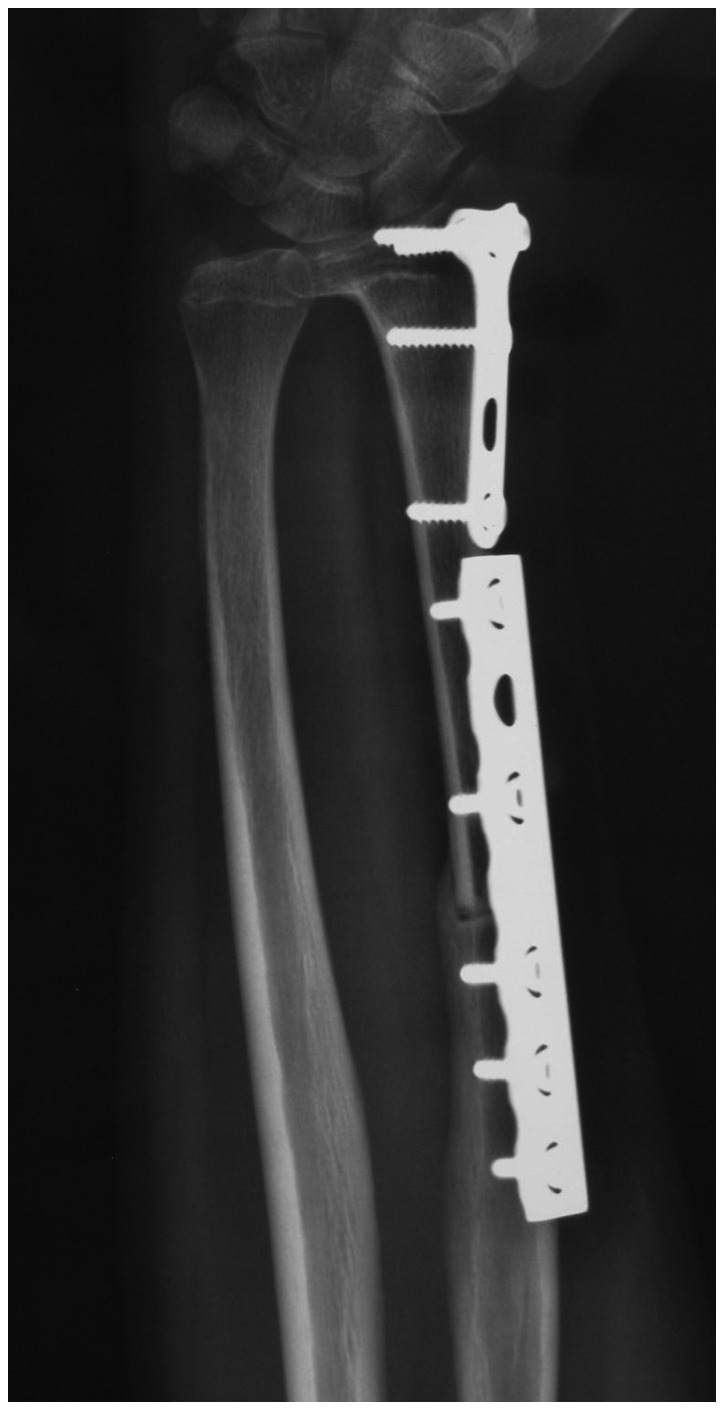
Six months after surgery, plain radiography revealed that the grafted fibular bone had healed well to the host bone.

**Figure 6:**
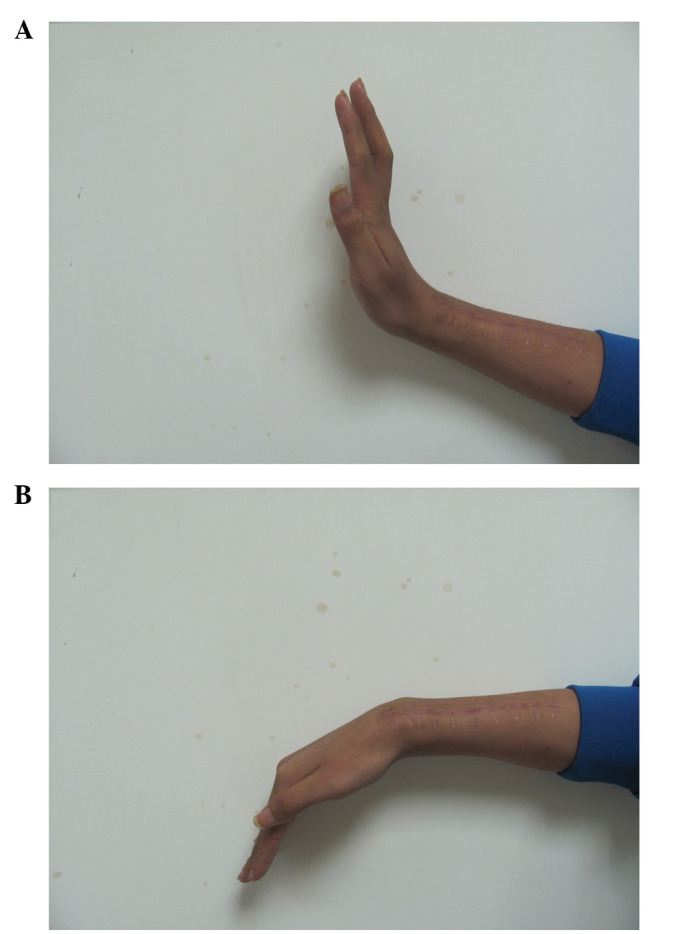
Physical examination of the affected wrist showed (A) active dorsiflexion to 90° and (B) palmer flexion to 45°.

The patient underwent en bloc resection of the tumor and reconstruction with a free fibular shaft to preserve the radiocarpal joint. A longitudinal dorsal incision at the radiocarpal joint was used to approach the distal radius and an elliptical excision was made at the needle biopsy site. The extensor tendons were removed and preserved, and the flexor tendons were preserved. A 13×3-cm osteotomy was performed proximal to the radial styloid followed by en bloc resection of the distal radial osteosarcoma (Fig. 3). The ulna and distal radioulnar articulation and radiocarpal joint were preserved, the free fibular shaft was fixed to the host bone with two plates (Fig. 4) and the wound was closed. A long arm cast was applied and the wrist was fixed in a functional position. The incision healed with no complications. Postoperative histological examination of the specimens revealed no tumor cells at the edges of the resected segment or in other regions of the lesion. Two weeks after surgery, chemotherapy with the same drug and dose as the preoperative protocol was administered and completed following six courses as the patient responded well. Progressive passive exercise was initiated once the affected distal radius and the wrist had been protected (by the plaster cast) for 12 weeks. Six months after surgery, radiographs revealed that the grafted fibular bone had healed well with the host bone (Fig. 5). Physical examination showed active dorsiflexion of the affected wrist was to 90° and wrist palmer flexion was to 45° (Fig. 6). One month after surgery, there was no evidence of wrist deformity, instability, metastasis or local recurrence. Further follow-up examinations are currently being conducted. Consent was obtained from both the patient and the patient’s family.

## Discussion

Various reconstructive procedures following the excision of malignant tumors in long bones have been reported, including prosthetic replacement, allografts, vascularized fibular grafts, autoclaved bone grafts and reimplantation of autologous inactivated bone (4–10). Generally, reconstructive procedures are selected depending on the site of tumor growth, effectiveness of preoperative chemotherapy and predicted limb function.

The distal radius is a relatively common skeletal site for primary bone tumors, however, not for osteosarcomas; it has been reported that <1% of osteosarcomas arise in the distal radius (2). Previous studies have reported en bloc resection of tumors and reconstruction with prosthesis, and non-vascularized or free proximal fibular grafting to treat giant cell tumors of the distal radius. Natarajan *et al* (9) reported 24 cases of aggressive benign and malignant tumors of the distal radius treated by resection and prosthetic replacement. Giant cell tumors were identified in 16 patients and osteosarcomas in eight. The mean Musculoskeletal Tumor Society (MSTS) functional score was 75% with a mean follow-up period of 78 months. The 10-year prosthesis survival rate was 87.5% and infection was the most common complication. Saini *et al* (11) investigated en bloc excision and reconstruction with ipsilateral non-vascularized fibula to treat aggressive giant cell tumors of the distal radius. The mean follow-up period was 5.8 years, the mean time for union at the fibuloradial junction was 33 weeks (14–69 weeks) and the mean range of movements were 52° forearm supination, 37° forearm pronation, 42° of wrist palmer flexion and 31° of wrist dorsiflexion. Overall, the revised MSTS score averaged 91.38% (range, 76.67–93.33%) with five excellent, four good and three satisfactory results. There were no cases with graft-related complications or deep infections, three cases of wrist subluxation, two cases of non-union and one case of tumor recurrence.

For tumors located in the metaphysis or in contact with the epiphyseal line, limb salvage surgery, to preserve epiphysis or the native joint, is required (13). When joint preservation is possible, the final affected limb functional evaluation shows the most satisfactory results. However, the joint surface preserving method may be performed only in a limited number of patients who adequately respond to chemotherapy and have a tumor in areas allowing joint surface preservation. This report presents a 17-year-old male patient with osteosarcoma of the distal radius treated at the General Hospital of Jinan Military Commanding Region (Jinan, China) with en bloc resection of the tumor and reconstruction with a free fibular shaft to preserve the radiocarpal joint. This technique was performed as the patient exhibited a marked response to preoperative chemotherapy and showed no evidence of epiphyseal invasion on the MRI scan. No local recurrence or metastasis was observed 14 months after surgery. In the final follow-up the movement ranges were wrist palmer flexion of 45° and wrist dorsiflexion of 90°.

In conclusion, the present report demonstrated that en bloc resection of a tumor and reconstruction with a free fibular shaft to preserve the radiocarpal articulation appears to be a promising procedure to treat osteosarcoma of the distal radius, with no evidence of epiphyseal invasion following effective preoperative chemotherapy. This technique preserved the important structures and joint surfaces to maintain wrist stability and effective function. However, the long-term outcomes of this technique require further investigation.
